# SARS-CoV-2: Structure, Biology, and Structure-Based Therapeutics Development

**DOI:** 10.3389/fcimb.2020.587269

**Published:** 2020-11-25

**Authors:** Mei-Yue Wang, Rong Zhao, Li-Juan Gao, Xue-Fei Gao, De-Ping Wang, Ji-Min Cao

**Affiliations:** Key Laboratory of Cellular Physiology at Shanxi Medical University, Ministry of Education, Key Laboratory of Cellular Physiology of Shanxi Province, and the Department of Physiology, Shanxi Medical University, Taiyuan, China

**Keywords:** severe acute respiratory syndrome coronavirus 2, protein structure, antibodies, antiviral compounds, vaccines

## Abstract

The pandemic of the novel severe acute respiratory syndrome coronavirus 2 (SARS-CoV-2) has been posing great threats to the world in many aspects. Effective therapeutic and preventive approaches including drugs and vaccines are still unavailable although they are in development. Comprehensive understandings on the life logic of SARS-CoV-2 and the interaction of the virus with hosts are fundamentally important in the fight against SARS-CoV-2. In this review, we briefly summarized the current advances in SARS-CoV-2 research, including the epidemic situation and epidemiological characteristics of the caused disease COVID-19. We further discussed the biology of SARS-CoV-2, including the origin, evolution, and receptor recognition mechanism of SARS-CoV-2. And particularly, we introduced the protein structures of SARS-CoV-2 and structure-based therapeutics development including antibodies, antiviral compounds, and vaccines, and indicated the limitations and perspectives of SARS-CoV-2 research. We wish the information provided by this review may be helpful to the global battle against SARS-CoV-2 infection.

## General Information of SARS-CoV-2

### Current Situation of SARS-CoV-2 Epidemic

In December 2019, the World Health Organization (WHO) was informed about an outbreak of pneumonia in Wuhan, Hubei Province, China, and the etiology was not identified. On January 30, 2020, WHO declared that the severe acute respiratory syndrome coronavirus 2 (SARS-CoV-2) epidemic is a public health emergency of international concern (PHEIC). On February 11, 2020, the WHO officially named the current outbreak of coronavirus disease as Coronavirus Disease-2019 (COVID-19) (Sun P. et al., 2020) and the International Committee on Taxonomy of Viruses (ICTV) named the virus as SARS-CoV-2 (Hu B. et al., 2020). Data as received by WHO from national authorities by October 11, 2020, there were more than 37 million confirmed cases with COVID-19 and 1 million deaths. Globally, the United States, India, and Brazil are the three countries with the largest cumulative number of confirmed cases in the world (https://www.who.int/docs/default-source/coronaviruse/situation-reports/20201012-weekly-epi-update-9.pdf). The total cumulative number of confirmed cases have far exceeded the number during SARS period (Wang and Jin, 2020). After the emergence of SARS-CoV and MERS-CoV, SARS-CoV-2 is the third zoonotic human coronavirus of the century (Gralinski and Menachery, 2020).

### The Origin and Evolution of SARS-CoV-2

Bioinformatic analyses showed that SARS-CoV-2 had characteristics typical of coronavirus family. It belongs to the betacoronavirus 2B lineage (Lai et al., 2020). Early in the pneumonia epidemic in Wuhan, scientists obtained the complete genome sequences from five patients infected with SARS-CoV-2. These genome sequences share 79.5% sequence identity to SARS-CoV. Obviously, SARS-CoV-2 is divergent from SARS-CoV. It is considered to be a new betacoronavirus that infects human (Zhou P. et al., 2020). Scientists aligned the full-length genome sequence of SARS-CoV-2 and other available genomes of betacoronaviruses. Results indicate the closest relationship of SARS-CoV-2 with the bat SARS-like coronavirus strain BatCov RaTG13, with an identity of 96%. These studies suggest that SARS-CoV-2 could be of bat origin, and SARS-CoV-2 might be naturally evolved from bat coronavirus RaTG13 (Zhang C. et al., 2020; Zhou P. et al., 2020).

One study analyzed the genomes of SARS-CoV-2 and similar isolates from the GISATD and NCBI (Xiong C. et al., 2020). Results indicate that an isolate numbered EPI_ISL_403928 shows different genetic distances of the whole length genome and different phylogenetic trees, the coding sequences of spike protein (S), nucleoprotein (N), and polyprotein (P) from other SARS-CoV-2, with 4, 2, and 22 variations in S, N, and P at the level of amino acid residues respectively. The results show that at least two SARS-CoV-2 strains are involved in the outbreak (Xiong C. et al., 2020).

After aligning the coding sequences (CDSs) based on the protein alignments, open reading frame 8 (ORF8) and open reading frame 10 (ORF10) of SARS-CoV-2 are different from other viruses. However, most ORFs annotated from SARS-CoV-2 are conserved. The overall genomic nucleotides identity between SARS-CoV-2 and SARS-like coronavirus strain BatCov RaTG13 is 96%. Compared with other viruses, the divergence of SARS-CoV-2 at neutral site is 17%, much larger than previously assessed. The spike gene exhibits larger dS (synonymous substitutions per synonymous site) values than other genes, which could be caused either by natural selection that accelerates synonymous substitutions or by a high mutation rate. Researchers obtained 103 SARS-CoV-2 genomes to recognize the genetic variants (Tang X. et al., 2020). Among the 103 strains, a total of 149 mutations are identified and population genetic analyses indicate that these strains are mainly divided into two types. Results suggest that 101 of the 103 SARS-CoV-2 strains show significant linkage between the two single nucleotide polypeptides (SNPs). The major types of SARS-CoV-2 (L type and S type) are distinguished by two SNPs which locate at the sites of 8,782 and 28,144. L type accounts for 70% of the 103 strains and S type accounts for 30%, suggesting L type is more prevalent than the S type. However, S type is the ancestral version of SARS-CoV-2 (Tang X. et al., 2020).

To date, 13 mutations in the spike protein have been identified. The mutation D614G should be paid special attention. In early February, the mutation Spike D614G began spreading in Europe. When introduced to new regions, it rapidly replaced the original strain to become the dominant strain (Korber B. et al., 2020). The D614G mutation in the spike protein would increase infectivity. S^G614^ is more stable than S^D641^ and less S1 shedding are observed, so the SARS-CoV-2 with S^G614^ could transmit more efficiently (Zhang et al., 2020b). One study shows that in multiple cell lines, the SARS-CoV-2 carrying the D614G mutation is eight times more effective at transducing cells than wild-type spike protein, providing evidence that the D614G mutation in SARS-CoV-2 spike protein could increase the transduction of multiple human cell types (Daniloski Z. et al., 2020). The D614G mutation could also decrease neutralization sensitivity to the sera of convalescent COVID-19 patients (Hu J. et al., 2020).

### The Epidemiological Characteristics of COVID-19

Bats appear to be the natural reservoir of SARS-CoV-2 (Zhang C. et al., 2020; Zhou P. et al., 2020). In one study, betacoronavirus isolated from pangolins has a sequence similarity of up to 99% with the currently infected human strain (Liu et al., 2020). Another study indicates that SARS-CoV-2 and the coronavirus from a pangolin in Malaysia has high genetic similarity. The gene similarity between these two viruses in terms of E, M, N, and S genes is 100, 98.6, 97.8, and 90.7%, respectively, suggesting the potential for pangolins to be the intermediate host (Xiao et al., 2020). Among the animals in close contact with humans, dogs, chickens, ducks, and pigs are not permissive to infection. SARS-CoV-2 replicates efficiently in cats and ferrets (Shi J. et al., 2020). SARS-CoV-2 can also transmit in golden hamster (Sia et al., 2020).

SARS-CoV-2 is transmitted *via* fomites and droplets during close unprotected contact between the infected and uninfected. Symptomatic and asymptomatic patients are the main source of infection. The virus can also spread through indirect contact transmission. Virus-containing droplets contaminate hands, people then contact the mucous membranes of the mouth, nose, and eyes, causing infection. The transmission of SARS-CoV-2 is not limited to the respiratory tract (Du et al., 2020). Some studies have demonstrated the aerosol transmission of SARS-CoV-2. During the COVID-19 outbreak, one study investigated the aerodynamic nature of SARS-CoV-2 by measuring viral RNA in aerosols in two Wuhan hospitals, indicating that SARS-CoV-2 has the potential to spread through aerosols. There may be a possibility of airborne transmission in health care facilities due to aerosols generated by medical procedures. Of note, in the spread of COVID-19, airborne transmission is the dominant route. (Chan et al., 2020; Meselson, 2020; Morawska and Cao, 2020; Sommerstein et al., 2020; Tang S. et al., 2020; van Doremalen et al., 2020; Zhang R. et al., 2020). In some pediatric SARS-CoV-2 infection cases, although children’s nasopharyngeal swabs are negative, rectal swabs are consistently positive, indicating the possibility of fecal-oral transmission (Xu et al., 2020). Recent studies demonstrate that SARS-CoV-2 could replicate effectively in human intestinal organoids and intestinal epithelium. As a result, SARS-CoV-2 has the potential to spread through intestinal tract. SARS-CoV-2 can also infect the intestinal cells of bats (Lamers et al., 2020; Zhou J. et al., 2020). A COVID-19 patient’s urine also contains infectious SARS-CoV-2 (Sun J. et al., 2020). After studying COVID-19 infection in nine pregnant women, the result suggests that there is no evidence that pregnant women who were infected SARS-CoV-2 in late pregnancy can transmit the virus to infant through intrauterine vertical transmission (Chen N. et al., 2020). However, recently, some studies demonstrated the possibility of vertical transmission of SARS-CoV-2 (Chen H. et al., 2020; Deniz and Tezer, 2020; Egloff et al., 2020; Hu X. et al., 2020; Mahyuddin et al., 2020; Oliveira et al., 2020; Parazzini et al., 2020; Peyronnet et al., 2020; Vivanti et al., 2020; Yang and Liu, 2020). In one case, the newborn whose mother was diagnosed with SARS-CoV-2 in the last trimester was infected with SARS-CoV-2, with neurological compromise. In another case, the cytokine levels and anti-SARS-CoV-2 IgM antibodies of the neonate is higher than normal, with no physical contact, suggesting the possibility of transplacental transmission (Dong et al., 2020). The risk of perinatal transmission of SARS-CoV-2 is relatively low. Compared with SARS-CoV-2, pregnant women infected with SARS and MERS showed more severe symptoms, such as miscarriage and abortion (Fan et al., 2020; Parazzini et al., 2020). According to current reports, the perinatal transmission can occur but the rate is low and the information about exposition during the first or second trimester of pregnancy remains unknown (Egloff et al., 2020; Parazzini et al., 2020). The major spread route of SARS-CoV-2 is person-to-person, it could happen in family, hospital, community, and other gathering of people. Most cases of the person-to-person transmission of the early stage in China happened in family clusters (Chan et al., 2020; Ghinai et al., 2020a; Ghinai et al., 2020b). This kind of spreading has the possibility to occur during the incubation period (Yu P. et al., 2020). It is worth noting that SARS-CoV-2 has high transmissibility during asymptomatic period or mild disease (Hu B. et al., 2020; Li et al., 2020). SARS-CoV-2 can also transmit from human to animal. Some animals, such as tiger, dog, and cat, are found to be infected with the virus through close contact with the infected people (Singla et al., 2020). A 17-years-old dog in Hong Kong was affected and it was the first case of human-to-animal transmission (https://www.afcd.gov.hk/english/publications/publi cationspress/pr2342.html). One study shows that the viral genetic sequences of SARS-CoV-2 detected in two dogs are the same with the SARS-CoV-2 in the corresponding human cases, suggesting the human-to-animal transmission. However, it remains unknown whether infected dogs can transmit the virus back to humans (Sit et al., 2020). SARS-CoV-2 is believed to transmit from the animal kingdom to human. According to the sequence analysis, bats are natural hosts for SARS-CoV-2 (Cui et al., 2019; Salata et al., 2019). SARS-CoV-2 and the coronavirus from a pangolin in Malaysia have high genetic similarity (Xiao et al., 2020), and the CoVs isolated from pangolins have the highest closeness to SARS-CoV-2 (Zhang T. et al., 2020), suggesting the potential for pangolins to be the intermediate host. The intermediate hosts could transmit the virus to susceptible people, leading to the newly appear diseases in humans (Ye et al., 2020; Zhang T. et al., 2020). SARS-CoV-2 can also transmit between animals. SARS-CoV-2 infected cats could transmit the virus to naïve cats with close contact (Halfmann et al., 2020). SARS-CoV-2 could also transmit in naïve ferrets, through direct or indirect contact (Kim et al., 2020).

According to current observed epidemiologic characteristics, everyone is considered susceptible and the median age is about 50 years (Chen N. et al., 2020; Guan et al., 2020; Huang et al., 2020; Wang D. et al., 2020; Wu and McGoogan, 2020).

The clinical manifestations differ with age. One study indicates that the cases over 60 years old have higher levels of blood urea nitrogen, inflammatory indicators, and more lobes bilateral lesions. The patients older than 60 years old have a greater chance of respiratory failure and longer disease courses. However, in those under 60, the severity is milder (Liu et al., 2020). One study reports a total of 72,314 confirmed cases in China, the majority of the patients (87%) are between the ages of 30 and 79. In the group no older than nine, no deaths occurred. However, in the group aged 70−79 years, the case-fatality rate (CFR) is 8.0%, in the group aged 80 years and older, the CFR is 14.8%. As to the patients with different comorbid conditions, such as cardiovascular disease, diabetes, chronic respiratory disease, hypertension, and cancer, the CFR is 10.5, 7.3, 6.3, 6.0, and 5.6%, respectively. These results suggest that comorbid conditions are high risk factors for COVID-19 patients and higher fatality rates are observed than those without underlying diseases (Wu and McGoogan, 2020). Among the 1,099 cases confirmed with COVID-19, patients with severe disease were 7 years older than those with non-severe disease (Guan et al., 2020). Of the 1,391 infected children, the median age is 6.7 years and most children show milder symptoms (non-pneumonia or mild pneumonia) than adults (Lu X. et al., 2020). The patients who aged ≥65 years old have a higher risk of mortality from COVID-19, especially the patients with acute respiratory distress syndrome (ARDS) and comorbidities (Du et al., 2020; Wu C. et al., 2020; Yang X. et al., 2020; Zhou F. et al., 2020).

### Clinical Characteristics of COVID-19

The most common manifestations of COVID-19 are fever and dry cough. The majority of the patients showed bilateral pneumonia. Old males with comorbidities are more likely to be affected by SARS-CoV-2 (Chen N. et al., 2020). The blood counts of patients showed leucopenia and lymphopenia. The content of IL2, IL7, IL10, GSCF, IP10, MCP1, MIP1A, and TNFα in the plasma of ICU patients is higher than non-ICU patients (Huang et al., 2020).

COVID-19 is divided into three levels according to the severity of the disease: mild, severe, and critical. The majority of patients only have mild symptoms and recover (Hu B. et al., 2020). Asymptomatic infection cases were also reported, but most of the asymptomatic patients went on to develop disease since the data of identification (Huang et al., 2020). **Table 1** shows the clinical manifestations of COVID-19 (Chen T. et al., 2020; Hu B. et al., 2020; Huang et al., 2020; Wang Y. et al., 2020; Wu and McGoogan, 2020) and three different levels of COVID-19 divided according to the severity (Chen T. et al., 2020; Hu B. et al., 2020; Huang et al., 2020; Wang Y. et al., 2020; Wu and McGoogan, 2020). Besides respiratory illness, COVID-19 disease could lead to myocardial injury and arrhythmic complications (Bansal, 2020; Kochi et al., 2020), neurological complications, such as myalgia, headache, dizziness, impaired consciousness, intracranial hemorrhage, hypogeusia, and hyposmia (Berger, 2020; Paybast et al., 2020), and even stroke (Hess et al., 2020; Trejo-Gabriel-Galán, 2020). Digestive symptoms and liver injury (Lee et al., 2020), hypercoagulability and thrombotic complications (Haimei, 2020) have also been reported. Critical patients could quickly progress to ARDS, hard-to-correct metabolic acidosis, septic shock, coagulation dysfunction, and multiple organ functional failure. Severe complications included ARDS, RNAaemia (detectable serum SARS-CoV-2 viral load), multiple organ failure, and acute cardiac injury. About 26.1% patients were admitted to the ICU because of complications caused by COVID-19 (Huang et al., 2020). With proper diagnosis and treatments for COVID-19, most patients had a good prognosis (Wang Y. et al., 2020). The elderly and the patients with underlying diseases have worse prognosis (Deng and Peng, 2020).

**Table 1:** Clinical manifestations and three different levels of COVID-19.

Clinical manifestations	fever, dry cough, fatigue, shortness of breath, muscle ache, confusion, headache, sore throat, rhinorrhea, chest pain, diarrhea, nausea, vomiting, chills, sputum production, haemoptysis, dyspnea, bilateral pneumonia anorexia, chest pain, leucopenia, lymphopenia, olfactory and taste disorders, higher levels of plasma cytokines (IL2, IL7, IL10, GSCF, IP10, MCP1, MIP1A, and TNFα) (ICU patients)	
Three different levels of COVID-19	Mild	fever, cough, fatigue, ground-glass opacities, non-pneumonia, and mild pneumonia
	Severe	dyspnea, blood oxygen saturation ≤93%, respiratory frequency ≥30/min, partial pressure of arterial oxygen to fraction of inspired oxygen ratio <300, and/or lung infiltrates >50% within 24 to 48 h, ICU needed
	critical	acute respiratory distress syndrome (ARDS), respiratory failure, septic shock, and/or multiple organ dysfunction or failure, hard-to-correct metabolic acidosis, septic shock, coagulation dysfunction

## The Structure of SARS-CoV-2

Coronaviruses belongs to the subfamily *Coronavirinae* in the family of Coronaviridae and the subfamily contains four genera: *Alphacoronavirus*, *Betacoronavirus*, *Gammacoronavirus*, and *Deltacoronavirus*. The genome of CoVs (27–32 kb) is a single-stranded positive-sense RNA (+ssRNA) which is larger than any other RNA viruses. The nucleocapsid protein (N) formed the capsid outside the genome and the genome is further packed by an envelope which is associated with three structural proteins: membrane protein (M), spike protein (S), and envelope protein (E) (Brian and Baric, 2005). As a member of coronavirus family, the genome size of SARS-CoV-2 which was sequenced recently is approximately 29.9 kb (Lu R. et al., 2020). SARS-CoV-2 contains four structural proteins (S, E, M, and N) and sixteen non-structural proteins (nsp1−16). Nsp1 mediates RNA processing and replication. Nsp2 modulates the survival signaling pathway of host cell. Nsp3 is believed to separate the translated protein. Nsp4 contains transmembrane domain 2 (TM2) and modifies ER membranes. Nsp5 participates in the process of polyprotein during replication. Nsp6 is a presumptive transmembrane domain. The presence of nsp7 and nsp8 significantly increased the combination of nsp12 and template-primer RNA. Nsp9 functions as an ssRNA-binding protein. Nsp10 is critical for the cap methylation of viral mRNAs. Nsp12 contains the RNA-dependent RNA polymerase (RdRp), which is a critical composition of coronavirus replication/transcription. Nsp13 binds with ATP and the zinc-binding domain in nsp13 participates in the process of replication and transcription. Nsp14 is a proofreading exoribonuclease domain. Nsp15 has Mn(2+)-dependent endoribonuclease activity. Nsp16 is a 2’-O-ribose methyltransferase (Naqvi et al., 2020). One study shows that there are some NSP-mediated effects on splicing, translation, and protein trafficking to inhibit host defenses. Upon SARS-CoV-2 infection, NSP16 binds mRNA recognition domains of the U1 and U2 snRNAs to suppress mRNA splicing. NSP1 binds to 18S ribosomal RNA in the mRNA entry channel of the ribosome to interfere with the translation of mRNA. NSP8 and NSP9 binds to the 7SL RNA which locates at the Signal Recognition Particle to disrupt protein trafficking to the cell membrane (Banerjee et al., 2020). Followings are some SARS-CoV-2 proteins which may potentially serve as antiviral drug targets based on their structures.

### Spike Glycoprotein

**Figure 1:**
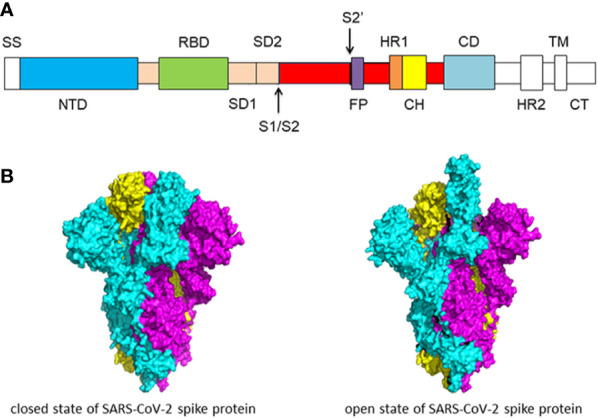
**(A)** Schematic of SARS-CoV-2 spike protein primary structure. Different domains are shown by different colors. SS, single sequence; NTD, N-terminal domain; RBD, receptor-binding domain; SD1, subdomain 1; SD2, subdomain 2; S1/S2, S1/S2 protease cleavage site; S2’, S2’ protease cleavage site; FP, fusion peptide; HR1, heptad repeat 1; CH, central helix; CD, connector domain; HR2, heptad repeat 2; TM, transmembrane domain; CT, cytoplasmic tail. The protease cleavage site is indicated by arrows. **(B)** Cryo-EM structure of the SARS-CoV-2 spike protein. The closed state (PDB: 6VXX) of the SARS-CoV-2 S glycoprotein (left) the open state (PDB: 6VYB) of the SARS-CoV-2 S glycoprotein (right).

The coronaviruses entry into host cells is mediated by spike glycoprotein (S protein) (Li et al., 2003; Li et al., 2005; Li, 2016). The transmembrane spike glycoproteins form homotrimers that protrude from the viral surface. The spike glycoprotein is critical for the entry of the coronaviruses so it is an attractive antiviral target. S protein is composed of two functional subunits, including the S1 and S2 subunits. The S1 subunit consists of N-terminal domain (NTD) and receptor binding domain (RBD). The function of S1 subunit is bind to the receptor on host cell. S2 subunit contains fusion peptide (FP), heptad repeat 1 (HR1), central helix (CH), connector domain (CD), heptad repeat 2 (HR2), transmembrane domain (TM), and cytoplasmic tail (CT) (**Figure 1A**). The function of S2 subunit is to fuse the membranes of viruses and host cells. The cleavage site at the border between the S1 and S2 subunits is called S1/S2 protease cleavage site. For all the coronaviruses, host proteases cleave the spike glycoprotein at the S2’ cleavage site to activate the proteins which is critical to fuse the membranes of viruses and host cells through irreversible conformational changes. N-linked glycans are critical for proper folding, neutralizing antibodies, and decorating the spike protein trimers extensively (Walls et al., 2020; Wrapp et al., 2020).

Overall, the structure of SARS-CoV-2 S protein resembles the closely related SARS-CoV S protein. In the prefusion conformation, S1 and S2 subunits remain non-covalently bound. Different kinds of coronaviruses utilize special domains in the S1 subunit to recognize different entry receptors. In the case of SARS-CoV and SARS-CoV-2, to enter host cells, they recognize the receptor angiotensin-converting enzyme 2 (ACE2) on host cells *via* the receptor binding domain (RBD). The S protein has two forms of structure, including the closed state and the open state (**Figure 1B**). In the closed state, the three recognition motifs do not protrude from the interface formed by three spike protein protomers. In the open state, the RBD is in the “up” conformation. The open state is necessary for the fusion of the SARS-CoV-2 and the host cell membranes, thereby facilitating SARS-CoV-2 to enter the host cells (Walls et al., 2020).

#### HR1 and HR2

The six-helical bundle (6-HB) is formed by HR1 and HR2 and is critical for membrane fusion which is dominated by the spike protein of SARS-CoV or SARS-CoV-2, making HR1 and HR2 an attractive drug target (Liu et al., 2004; Xia et al., 2020b). The difference between the 6-HB of SARS-CoV-2 and SARS-CoV may stabilize 6-HB conformation of SARS-CoV-2 and enhance the interactions between HR1 and HR2, resulting in the increased infectivity of SARS-CoV-2. The HR1-L6-HR2 complex contains most parts of HR1 and HR2 domain and a linker (Xia et al., 2020a). This fusion protein exhibits a rod-like shape and it is the standard structure of 6-HB. Three HR1 domains come together to form a spiral coil trimer in a parallel manner. Three HR2 domains are entwined around the coiled-coil center in an antiparallel manner which is mainly mediated by hydrophobic force. Hydrophobic residues on theHR2 domian binds with the hydrophobic groove formed by every two two neighboring HR1 helices. The overall 6-HB structure of SARS-CoV and SARS-CoV-2 is very similar, especially the S2 subunit (Xia et al., 2020a). The identity of the HR1 of SARS-CoV and SARS-CoV-2 is 96% and HR2 is 100%. There are eight distinct residues in the fusion core region of HR1 domain. In the HR1 domain of SARS-CoV, lysine 911 binds to the glutamic acid 1176 in HR2 domain through a salt bridge. As to SARS-CoV-2, the salt bridge is replaced by a strong hydrogen bond between serine 929 in HR1 and serine 1,196 in HR2. In SARS-CoV HR1, glutamine 915 has no interaction with HR2. However, as to SARS-CoV-2, there is a salt bridge between lysine 933 in HR1 and asparagine 1,192 in HR2 (Xia et al., 2020a). In SARS-CoV, there is a weak salt bridge between glutamic acid 918 in HR1 and arginine 1,166. However, aspartic acid 936 in the HR1 of SARS-CoV-2 binds to the arginine 1,158 through a salt bridge. In the SARS-CoV, lysine 929 binds to the glutamic acid 1,163 in the HR2 domain through a salt bridge and threonine 925 does not bind to the glutamic acid 1,163. However, serine 943 and lysine 947 in the SARS-CoV-2 bind to the glutamic acid 1,182 in HR2 through a hydrogen bond and a salt bridge. These differences may result in increased infectivity of SARS-CoV-2 (Xia et al., 2020a).

#### The Receptor Binding Domain (RBD)

The spike protein of SARS-CoV-2 contains an RBD that recognizes the receptor ACE2 specifically. RBD is a critical target for antiviral compounds and antibodies (Letko et al., 2020). SARS-CoV-2 RBD includes two structural domains: the core and the external subdomains. The core subdomain is highly conserved. It is composed of five β strands arranged in antiparallel manner and a disulfide bond between two β strands. The external subdomain is mainly dominated by the loop which is stabilized by the disulfide bond (Wang Q. et al., 2020). The SARS-CoV-2 RBD core consists of five β sheets arranged in antiparallel manner and connected by loops and short helices. Between the antiparallel β4 and β7 strands is the receptor-binding motif (RBM) which consists of loops and α helices, as well as short β5 and β6 strands. RBM contains most binding sites for SARS-CoV-2 and ACE2. Eight of the nine Cys residues in the RBD form four pairs of disulfide bonds. Three disulfide bonds are in the core of RBD, enhancing the stabilization of the β sheet (C336-C361, C379-C432, and C391-C525). With respect to the remaining disulfide bond (C480-C488), it promotes the connections between the loops in RBM. The peptidase domain in the N-terminal of ACE2 contains the binding site, which is formed by two lobes of RBM and ACE2. RBM binds to the small lobe of the ACE2 on the bottom side. The surface of RBM is slightly concave inward to make room for ACE2 (Lan et al., 2020).

One study obtained a 3.5 Å-resolution structure of spike protein trimer with one RBD in the in the “up” conformation (receptor-accessible state). Receptor binding destabilizes the prefusion structure, triggered by this process, the S1 subunit dissociates and the S2 subunit refolds into a stable postfusion conformation, which has been captured in SARS-CoV. RBD goes through conformational transitions like a hinge, leading to the hide or exposure of the determinants of the spike protein to engage a host cell receptor. This process will form the following two states: “down” conformation and “up” conformation. In the “down” conformation, SARS-CoV-2 could not recognize the ACE2 on the host cells. The structure of SARS-CoV-2 is highly similar with SARS-CoV. One of the larger differences is in the down conformation, SARS-CoV RBD packs tightly against the NTD of the neighboring protomer, while the angle of SARS-CoV-2 RBD is near to the central cavity of the spike protein trimer. When aligned the individual structural domains corresponding to SARS-CoV-2 and SARS-CoV, highly similar structures were observed (Wrapp et al., 2020). The overall structure of SARS-CoV-2 RBM is also nearly identical to that identified in previous studies, with only one observed difference on the distal end (Lan et al., 2020).

#### RBD-ACE2 Complex

**Figure 2:**
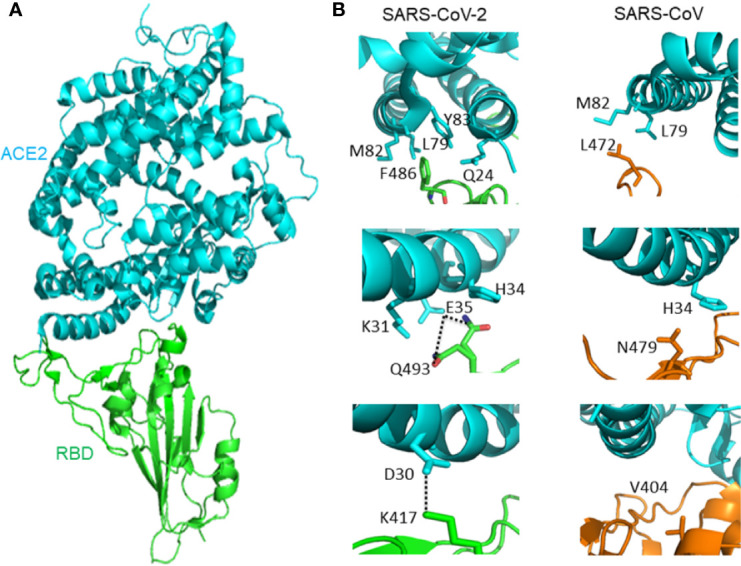
**(A)** The overall structure of SARS-CoV-2 RBD bound with ACE2. ACE2 is colored cyan, SARS-CoV-2 RBD core is colored green (PDB: 6M0J). **(B)** Different interactions between SARS-CoV-2 RBD/ACE2 (PDB: 6M0J) and SARS-CoV RBD/ACE2 (PDB: 2AJF) that contribute to binding affinity difference. ACE2 is colored cyan. The RBD of SARS-CoV-2 is green, and the RBD of SARS-CoV is orange. Hydrogen bond between Q493 and E35 is represented by dash lines. Salt-bridge between ACE2 D30 and SARS-CoV-2 K417 is represented by dash lines.

It is important to understand the receptor recognition mechanism of the SARS-CoV-2, which determines the infectivity, host range, and pathogenesis of the virus. Both SARS-CoV-2 and SARS-CoV recognize the ACE2 in humans (Li et al., 2003; Li et al., 2005; Sia et al., 2020). The crystal structure of SARS-CoV-2 RBD bound with ACE2 has been determined (**Figure 2A**). The overall combination mode of SARS-CoV-2 RBD-ACE2 complex is highly similar with that of the identified SARS-CoV RBD-ACE2 complex in previous study. Seventeen of the 20 residues of the ACE2 interacting with the RBD of SARS-CoV and SARS-CoV-2 are the same.

However, there are subtle distinct ACE2 interactions which lead to the variation in binding affinity between SARS-CoV-2 and SARS-CoV RBD to ACE2. The affinity between ACE2 and SARS-CoV-2 is higher than the affinity between ACE2 and SARS-CoV. At the F486/L472 position, SARS-CoV-2 F486 interacts with ACE2 Q24, L79, M82, and Y83, and SARS-CoV L472 only interacts with ACE2 L79 and M82. At the Q493/N479 position, SARS-CoV-2 Q493 interacts with ACE2 K31, E35, and H34. There is a hydrogen bond between Q493 and E35. SARS-CoV N479 only interacts with ACE2 H34. Outside SARS-CoV-2 RBM, there is a salt bridge between ACE2 D30 and SARS-CoV-2 K417. However, the SARS-CoV V404 failed to participate in ACE2 binding (Lan et al., 2020) (**Figure 2B**).

Another study shows the crystal structure of chimeric SARS-CoV-2 RBD-ACE2 complex. The constructed chimeric RBD which contains the RBM of SARS-CoV-2 as the function-related unit and the SARS-CoV RBD core as the crystallization scaffold could facilitate crystallization. The side loop from SARS-CoV-2 (away from the main binding interface) maintains a salt bridge between RBD R426 and ACE2 E329. This side loop could further facilitate crystallization. The structure of chimeric RBD-ACE2 complex is highly similar with the wild-type RBD-ACE2 complex as introduced above, especially in the RBM region. SARS-CoV-2 RBM forms a surface which is gently concave, binding to the claw-like structure on the exposed outer surface of ACE2. There is a N-O bridge between R439 of the chimeric RBD and E329 of ACE2. The N-O bridge is non-natural, resulting from the SARS-CoV-based chimaera. The binding affinity between chimeric RBD and ACE2 is higher than the binding affinity between wild-type SARS-CoV-2 RBD and ACE2. It is obvious that the ACE2-binding affinity of SARS-CoV RBD is lower than SARS-CoV-2 and chimeric RBDs (Shang et al., 2020).

#### Furin Cleavage Site of the Spike Protein

The S1/S2 boundary of SARS-CoV-2 spike protein constitutes the cleavage site for the subtilisin-like host cell protease furin, which sets SARS-CoV-2 S apart from SARS-CoV S. The furin cleavage site includes four residues (P681, R682, R683, and A684) and is located at the boundary between the S1 and S2 subunit. Functionally, R682, R683, A684, and R685 constitute the minimal polybasic furin cleavage site, RXYR, where X or Y is a positively charged arginine or lysine (Li, 2020). Such polybasic cleavage sites are not present in SARS-CoV and SARS-CoV-related group 2b betacoronaviruses found in humans, which may contribute to the high virulence of SARS-CoV-2 as a result of furin proteases required for proteolytic activation of S are ubiquitously expressed in humans, providing expanded tissue tropism and pathogenesis (Sternberg and Naujokat, 2020).

Additionally, a study has generated a SARS-CoV-2 mutant virus lacking the furin cleavage site (δPRRA) in the spike protein. The mutant virus had reduced spike protein processing in Vero E6 cells as compared to wild type SARS-CoV-2 virus. The mutant virus also had reduced replication in Calu3 human respiratory cells and had attenuated disease in a hamster pathogenesis model. These results showed an important role of the furin cleavage site in SARS-CoV-2 replication and pathogenesis (Johnson et al., 2020).

### The RNA-Dependent RNA Polymerase (RdRp)

**Figure 3:**
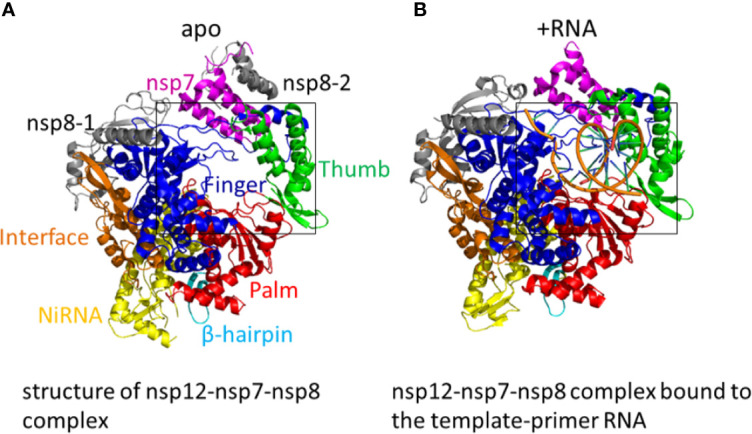
**(A)** The structure of nsp12-nsp7-nsp8 complex. Color marks: nsp7, magenta; nsp8-1 and nsp8-2, grey; β-hairpin, cyan; NiRAN, yellow; the interface, orange; the fingers domain, blue; the palm domain, red; the thumb domain, green. (PDB: 6M71) **(B)** The structure of the SARS-CoV-2 RNA-dependent RNA polymerase (RdRp) in active form. The nsp12-nsp7-nsp8 complex bound to the template-primer RNA. (PDB: 7BV2).

The replication of SARS-CoV-2 is dominated by a replication/transcription complex which contains several subunits. The complex is composed of viral non-structural proteins (nsp) and the core of the complex is the RdRp in nsp12. The functions of the nsp12 require accessory factors, including nsp7 and nsp8. Nsp12 alone has little activity. The presence of nsp7 and nsp8 significantly increased the combination of nsp12 and template-primer RNA. The crystal structure of nsp12-nsp7-nsp8 complex has been identified (**Figure 3A**). RNA-dependent RNA polymerase, which catalyzes the synthesis of viral RNA, is a critical composition of coronavirus replication/transcription. RdRp is an important antiviral drug target. The structures on the SARS-CoV-2 nsp12 contain a nidovirus-unique N-terminal extension domain which adopts a nidovirus RdRp-associated nucleotidyltransferase (NiRAN) structure and a “right hand” RNA-dependent RNA polymerase domain in the C-terminal. These two domains are connected by an interface domain. A unique β-hairpin is observed in the N-terminal extension domain. The β-hairpin forms close contacts to stabilize the overall structure. The RNA-dependent RNA polymerase domain contains three subdomains: a fingers subdomain, a palm subdomain, and a thumb subdomain. The β-hairpin structure inserts into the clamping groove formed by the palm subdomain and the NiRAN domain. In the plam domain, polymerase motifs A−G which is highly conserved form the active site chamber of SARS-CoV-2 RdRp domain. The RdRp motifs mediate template-directed RNA synthesis in a central cavity through four positively charged solvent-accessible paths, including template entry path, primer entry path, the NTP entry channel, and the nascent strand exit path (Gao Y. et al., 2020). A recent study shows the cryo-electron microscopic structure of the nsp12-nsp7-nsp8 complex in active form (Yin et al., 2020) (**Figure 3B**).

When added a minimal RNA hairpin substrate, the complex nsp12-nsp7-nsp8 exhibited RNA-dependent RNA extension activity. The structure of RdRp-RNA complex shows nsp12-nsp7-nsp8 complex engaged with more than two turns of duplex RNA. The RdRp-RNA structure is similar to that of the free enzyme with some unique characteristics. Compared with free enzyme, the RdRp-RNA complex contains an extended protein region in nsp8 and a protruding RNA. The subunit nsp12 binds with the first turn of RNA between its thumb subdomains and fingers subdomains. The palm subdomain contains the active site which is formed by five nsp12 motifs A−E. Motif C interacts with the 3’ end of RNA and includes the aspartic acid 760 and 761. The nsp12 motifs F and G lies in the fingers subdomain and have the function of positioning the RNA template. As the RNA duplex leaves the cleft of the RdRp, it forms a second helical turn, protruding from the surface of nsp12. No structural factors in the RdRp will limit RNA duplex extension. Between the α-helical extensions is the RNA duplex. The N-terminal regions, which are located in the two nsp8 subunits and are highly conserved, form the α-helical extensions. These nsp8 extensions use the positively charged residues to interact with the RNA backbones. The nsp8 could function as the “sliding poles”, sliding along the protruding RNA to prevent RdRp from dissociating prematurely during replication. The triphosphate-binding site is conserved. Residues D623, S682, and N691 are likely to interacts with the 2’-OH group of the triphosphate (NTP), making the RdRp special for the synthesis of RNA instead of DNA (Hillen et al., 2020).

### The Main Protease

The main protease (M^pro^) of SARS-CoV-2 plays a pivotal role in mediating the replication and transcription of viral gene. M^pro^ hydrolyzes the polyprotein at least eleven conserved sites and begins with cleaving the pp1a and pp1b of M^pro^. Considering the absence of closely related homologues in humans, together with the functional importance of the main protease in the life cycle of the virus, the main protease is an attractive antiviral target. The crystallographic symmetry shows that M^pro^ forms a homodimer (protomer A and protomer B). Each protomer contains three subdomains, namely domain I, domain II, and domain III. A long loop connects domain II and domain III. The cleft between domain I and domain II lies the substrate-binding pocket, which features the catalytic dyad residues His41 and Cys145 (Jin et al., 2020a). As to all the coronaviruses, the active sites of M^pro^ are highly conserved and consists of four sites: S1’, S1, S2, and S4. In the S1’ site, the thiol of a cysteine anchors inhibitors by a covalent linkage. For inhibitors, the covalent linkage is critical to maintain its antiviral activity (Yang et al., 2005).

The spike protein is critical in the process of SARS-CoV-2 invading host cells. The main protease and RdRp have important functions in the replication of SARS-CoV-2. As a result, the spike protein, main protease, and RdRp are important anti-SARS-CoV-2 drug targets, providing ideas for the development of antibodies, drugs, and vaccines.

## Structure-Based Antibodies Against SARS-CoV-2

### Meplazumab

Recently, the study indicates that SARS-CoV-2 invades host cells through a new route: CD147-spike protein, through which spike protein bound to CD147, a transmembrane glycoprotein belongs to the immunoglobulin superfamily, thereby mediating the invasion of SARS-CoV-2. Meplazumab is an anti-CD147 humanized antibody. It could block CD147 and significantly prevent the SARS-CoV-2 from entering host cells. BIOcore experiment shows that the affinity constant between CD147 and RBD is 1.85×10^-7^ M. Unlike ACE2, CD147 is highly expressed in inflamed tissues, pathogen infected cells, and tumor tissues. It has low cross-reaction with normal cells. As a result, CD147 targeted drugs are safe and reliable (Wang K. et al., 2020).

### Monoclonal Antibody 4A8

One study isolated monoclonal antibodies (MAbs) from ten SARS-CoV-2 infected patients in recovery period. Among these antibodies, MAb 4A8, exhibits high neutralization activities against SARS-CoV-2. The crystal structure of spike protein-4A8 complex at 3.1 Å resolution shows that three 4A8 monoclonal antibodies binds the N-terminal domain (NTD) of the spike protein trimer. Each of the 4A8 monoclonal antibody interacts with one N-terminal domain (NTD) of the spike protein.

The crystal structure of spike protein-4A8 complex at 3.1 Å resolution shows that each one of the three 4A8 monoclonal antibodies binds to one N-terminal domain (NTD) of the spike protein trimer. The asymmertric conformation of the trimeric spike protein exhibits one of three RBD in “up” conformation and two RBDs in “down” conformation. The interface between 4A8 and the corresponding NTD is identical. Among the five new constructed loops for NTD which are designed as N1 which are designated as N1−N5, N3 and N5 loops dominate the interactions with 4A8. Three complementarity-determining regions (CDRs) which are designated as CDR1, CDR2, and CDR3 on the heavy chain of 4A8 binds with NTD. R246 on the N5 loop interacts with the Y27 and E31 of 4A8 on the CDR1. K150 and K147 on the N3 loop form salt bridges with E54 and E72 of 4A8 respectively. There are hydrogen bonds between K150 and 4A8-Y111, H146 and 4A8-T30 (Chi et al., 2020).

### Monoclonal Antibody 47D11

A human monoclonal antibody (mAb) 47D11 is found to potently block SARS-CoV-2 infection. The target of 47D11 is the RBD and spike ectodomain (S_ecto_) of the SARS-CoV-2 spike protein. The 47D11 binds to the RBD of SARS-CoV and SARS-CoV-2 with similar affinity constant. However, the binding affinity between 47D11 and SARS-CoV-2 S_ecto_ was lower than that of SARS-CoV. The binding of 47D11 to SARS-CoV-RBD and SARS-CoV-2-RBD did not compete with the binding of 47D11 to the ACE2 receptor on the cell surface (Wang C. et al., 2020). Despite the relatively high degree of structural similarity between the SARS-CoV RBD and the SARS-CoV-2 RBD, when using the three reported SARS-CoV RBD-directed monoclonal antibodies which have a strong binding to the SARS-CoV RBD, there is no detectable binding for any of the three mABs (S230, m396, 80R) at the tested concentration. Because of the different antigenicity, SARS-directed mAbs have no absolute cross reactions with SARS-CoV-2-directed mAbs (Wrapp et al., 2020).

### Monoclonal Antibody CR3022

**Figure 4:**
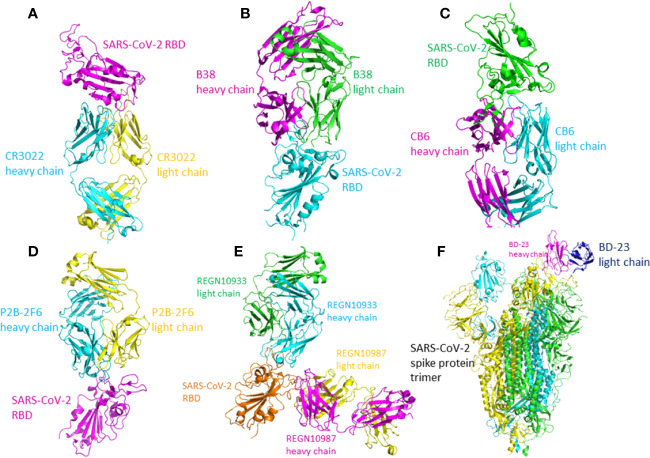
The crystal structure of the antibody-RBD/spike protein complex. **(A)** Crystal structure of CR3022 in complex with SARS-CoV-2 RBD. CR3022 heavy chain is colored in cyan and light chain in yellow. The SARS-CoV-2 RBD in colored in magenta (PDB: 6W41). **(B)** The crystal structure of B38/SARS-CoV-2 RBD. The heavy chain of B38 is colored magenta and the light chain is colored green. The RBD is colored cyan (PDB: 7BZ5). **(C)** The crystal structure of CB6-Fab/SARS-CoV-2-RBD. The heavy chain of CB6 is colored magenta and the light chain of CB6 is colored cyan. The SARS-CoV-2-RBD is colored green (PDB: 7C01). **(D)** The crystal structure of P2B-2F6 Fab/SARS-CoV-2 RBD complex. The light chain of P2B-2F6 Fab is colored yellow and the heavy chain is colored cyan. The SARS-CoV-2 RBD is colored magenta (PDB: 7BWJ). **(E)** The crystal structure of the complex of SARS-CoV-2 spike RBD bound to Fab fragments of REGN10933 and REGN10987. REGN10933 heavy and light chains are cyan and green, and REGN10987 heavy and light chains are magenta and yellow, respectively (PDB: 6XDG). **(F)** The crystal structure of BD-23 Fab/spike protein trimer complex. The light chain of BD-23 Fab is colored blue and the heavy chain is colored magenta. The three protomers in the spike protein trimer are colored cyan **(A)**, green **(B)**, and yellow **(C)** (PDB: 7BYR).

The SARS-CoV-speciﬁc antibody, which was discovered in the plasma of a SARS-infected patient in recovery period, CR3022, could also bind with the RBD of SARS-CoV-2 potently. After saturating the streptavidin biosensors which labelled with biotinylated SARS-CoV-2 RBD, followed by the mixture of CR3022 and ACE2, the results indicated that the binding sites of CR3022 on RBD is different from ACE2. The mixture of CR3022 and CR3014 (a potent SARS-CoV-specific neutralizing antibody) neutralized SARS-CoV-2 in a collaborative way, with different epitopes on RBD. In conclusion, CR3022 has the potential to function as one kind of therapeutics, alone or with other neutralizing antibodies (Tian et al., 2020). The crystal structure of CR3022-RBD complex has been determined (**Figure 4A**). The light chain, heavy chain, and six CDR loops (H1, H2, H3, L1, L2, and L3) of CR3022 are used to interact with the RBD of SARS-CoV-2.

CR3022’s recognition of SARS-CoV-2 is mainly mediated by hydrophobic interactions. As to SARS-CoV and SARS-CoV-2, 24 of 28 residues buried by antibody CR3022 are the same, which is the cause of the cross-reactivity of CR3022. Although the high similarities of sequence, the affinity between CR3022 and SARS-CoV RBD is much higher than the affinity between CR3022 and SARS-CoV-2 RBD, likely resulting from the non-conserved residues in the epitope. Only when the RBD is in the “up” conformation, the epitope of CR3022 is exposed. If only one RBD on the trimeric S protein is in the “up” conformation, there would exist some clashes between CR3022 and RBD to hinder the bind. First, the variable region of CR3022 collides with S2 subunit of RBD, as well as the adjacent RBD in “down” conformation. Second, the constant region of CR3022 collides with NTD. When the targeted-RBD are in the double-“up” conformation (at least two) with a slight rotation, the binding epitope of the RBD can be accessed by CR3022 and all the clashes can be resolved (Yuan et al., 2020).

### Monoclonal Antibodies B38 and H4

The monoclonal antibodies B38 and H4 isolated from a convalescent patient display neutralization ability. The crystal structure of B38-RBD complex has been identified (**Figure 4B**). B38 and H4 are able to hinder the binding between SARS-CoV-2 RBD and cellular receptor ACE2. The epitopes of B38 and H4 on the RBD are different. As a result, B38 and H4 has the potential to function as the noncompeting monoclonal antibody pair to treat COVID-19. In infected lungs, B38 and H4 can reduce virus titers. The crystal structures of RBD-B38 indicates that two CDRs on the light chain and all the three CDRs on the heavy chain of CR3022 interacts with RBD. A total of 21 amino acids in the RBD binds with the heavy chain and 15 residues with the light chain. Among the 36 residues, only 15 residues are conserved between SARS-CoV and SARS-CoV-2. Hydrophilic interactions mediate most of the contacts between B38 and RBD. Water molecules play a significant role in the binding between SARS-CoV-2 RBD and B38. The comparison of RBD/B38-Fab complex and RBD/hACE2 complex shows no obvious conformational changes and 18 of 21 amino acids on the RBD are conserved between B38 and ACE2. This explains why antibody B38 blocks SARS-CoV-2 from binding to receptor ACE2 (Wu et al., 2020b).

### Monoclonal Antibodies CA1 and CB6

Two human monoclonal antibodies CA1 and CB6 could potently neutralize the SARS-CoV-2 *in vitro*. Particularly, CB6 could reduce lung damage and inhibit the titer of SARS-CoV-2 in rhesus monkeys, thereby having the potential to treat and prevent SARS-CoV-2 infection. The crystal structure of CB6-Fab/SARS-CoV-2-RBD complex indicates that CB6 binds to the RBD of SARS-CoV-2 (**Figure 4C**). CDR1, CDR2, and CDR3 loops in the CB6 V_H_ dominate the interaction between the CB6 and SARS-CoV-2-RBD, forming concentrated hydrophobic interactions and polar contacts. CB6 light chain has limited interactions with SARS-CoV-2-RBD, with only one hydrogen bond between Y505 and Y92. The superimposition of CB6/SARS-CoV-2-RBD complex and hACE2/SARS-CoV2-RBD complex indicated the steric competition between hACE2 and CB6 for RBD binding. The steric hindrance caused by CB6 is dominated by both the light chain and heavy chain of CB6, thus resulting in structure clashes with the SARS-CoV-2-RBD. CB6 and hACE2 have many overlapping binding sites on the RBD. In conclusion, steric hindrance caused by the V_H_ and V_L_ of CB6 and the overlapped binding areas inhibit the binding of SARS-CoV-RBD and hACE2 (Shi R. et al., 2020).

### Monoclonal Antibody P2B-2F6

Total 206 kinds of RBD-specific monoclonal antibodies have been isolated from the B cells of 8 COVID-19 patients. The most potent neutralizing antibodies are P2C-1F11, P2B-2F6, and P2C-1A3. The crystal structure of P2B-2F6/SARS-CoV-2 RBD complex has been determined at 2.85 Å resolution (**Figure 4D**). The interactions between P28-2F6 and the SARS-CoV-2 RBD is dominated by the heavy chain of P28-2F6. The paratope contains 3 light chain residues and 14 heavy chain residues. All the 12 epitopes residues are in the RBM, including lysine 444, glycine 446 and 447, asparagine 448, tyrosine 449, asparagine 450, leucine 452, valine 483, glutamic acid 484, glycine 485, phenylalanine 490, and serine 494. At the binding interface, there are hydrophobic interactions between P2B-2F6 and SARS-CoV-2 RBD residues Y449, L452, and F490, facilitating P2B-2F6 attachment. Hydrophilic interactions also exit at the binding interface. Structural superimposition of SARS-CoV-2 RBD/ACE2 complex and SARS-CoV-2 RBD/P2B-2F6 complex shows that the light chain of P2B-2F6 clashes with ACE2 residues aspartic acid 67, lysine 68, alanine 71, lysine 74, glutamic acid 110, and lysine 114, inhibiting the binding of ACE2 and RBD. The residues in RBD recognized by both P2B-2F6 and ACE2 are Y449 and G446. Compared with the binding affinity between ACE2 and RDB, the binding affinity between P2B-2F6 and RBD is higher. P2B-2F6 Fab could connect with both the “up” and “down” conformations of the RBDs of the trimer spike protein, while ACE2 only binds the “up” conformation of RBD (Ju et al., 2020).

### Antibody Cocktail: REGN10987 and REGN10933

One study used both genetically modified mice and B cells from SARS-CoV-2 convalescent patients to collect monoclonal antibodies. It has been identified that REGN10987 and REGN10933 are a pair of highly potent individual antibodies. The epitope of REGN10933 is located at the top of the RBD while the epitope of REGN10987 is located at the side of the RBD. They can bind to the RBD of SARS-CoV-2 simultaneously without competition. As a result, REGN10987 and REGN10933 can be paired in a therapeutic antibody cocktail. The bind of REGN10933 to RBD overlap the binding site for ACE2 extensively. However, the binding of REGN10987 has no or little overlap with the binding site of ACE2. The crystal structure has been identified (Hansen et al., 2020) (**Figure 4E**).

### Monoclonal Antibody BD-23

High-throughput single-cell RNA and VDJ sequencing were used to identify SARS-CoV-2 neutralizing antibodies from the B cells of 60 convalescent patients and 14 antibodies with strong neutralization ability were discovered, including the neutralizing antibody BD-23. The crystal structure of BD-23-Fab/spike protein at 3.8 Å resolution has been solved (**Figure 4F**). The spike adopts an asymmetric conformation. Two RBDs of the spike protein trimer adopt “down” conformation and the other adopts “up” conformation. Structural superimposition of SARS-CoV-2 RBD/ACE2 complex and SARS-CoV-2 RBD/BD-23 complex shows that BD-23 could clash with ACE2 to inhibit the RBD-ACE2 binding, endowing BD-23 with the SARS-CoV-2 neutralizing ability (Cao et al., 2020).

A variety of other antibodies are found targeting the spike protein of the SARS-CoV-2. Antibody n3130 and n3088 target the S_RBD_ and S1 subunit with the affinity of 55.4 nM (Wu et al., 2020a). Antibody S309 comes from B cells of SARS rehabilitation patients. It has cross-reaction with SARS-CoV and the affinity with its target S^B^ is 0.1 nM. The crystal structure of S309 has been identified (Pinto et al., 2020). Antibodies n3103, n3088, and S309 do not block the binding of SARS-CoV-2 with its receptor ACE2. Horse F(ab’)2 comes from horse serum immunized with RBD and the affinity between F(ab’)2 and RBD is 0.76 nM (Pan et al., 2020).

## Structure-Based SARS-CoV-2 Inhibitors

Currently, some small-molecule compounds have been developed which showed inhibitory effects on the SARS-CoV-2 infection, as described below.

### Remdesivir

Remdesivir is an adenosine analogue and is a potent inhibitor of RdRp. Remdesivir could potently inhibit the replication of SARS-CoV-2 *in vitro*. Remdesivir shows broad-spectrum antiviral effects against RNA virus infection in cultured cells, nonhuman primate models, and mice. As an adenosine analogue, remdesivir functions after virus entry, *via* incorporating into nascent viral RNA to terminate the replication before the RNA become mature (Gurwitz, 2020). Remdesivir is a kind of prodrug. In target cells, it would transform into the triphosphate form (RTP) and become active (Siegel et al., 2017). Like other nucleotide analog prodrugs, remdesivir inhibits the RdRp activity through covalently binds the primer strand to terminate RNA chain. Upon adding ATP, the nsp12-nsp7-nsp8 complex exhibits the function of RNA polymerase. However, with the addition of the active triphosphate form of remdesivir (RTP), the RNA polymerization activity would be significantly inhibited. The structure of the apo RdRp is composed of nsp12, nsp7, and nsp8. Besides, the template-RTP RdRp complex is composed of a 14-base RNA in the template strand as well as 11-base RNA in the primer strand. Of note, the remdesivir is in the monophosphate form (RMP) in the complex. The RMP is covalently linked to the primer strand, three magnesium ions, and a pyrophosphate. The three magnesium ions locate near the active site and promote catalysis. The RMP locates in the catalytic active site center. The catalytic active site is composed of seven motifs. There are base-stacking interactions between RMP and the base of the primer strand in the upstream. Hydrogen bonds also exists between RMP and the uridine base of the template strand. There are also interactions between RMP and side chains (K545 and R555). Twenty-nine residues from nsp12 participate the binding of the RNA directly. No residue from nsp7 or nsp8 mediates the RNA interactions (Yin et al., 2020).

Similar to remdesivir, favipiravir is also an inhibitor of the RdRp. The structure of favipiravir resembles the endogenous guanine. Clinical trial demonstrated that favipiravir had little side effect as the first anti-SARS-CoV-2 compound conducted in China (Furuta et al., 2017; Tu et al., 2020).

### N3

**Figure 5:**
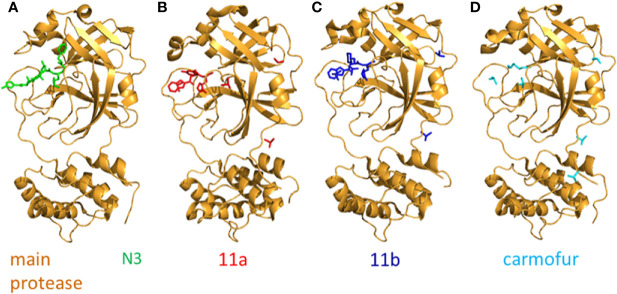
The crystal structure of N3 and its inhibitors. **(A)** The crystal structure of N3-main protease complex. The main protease is colored brightorange. N3 is colored green (PDB: 6LU7). **(B)** The crystal structure of 11a-main protease complex. The main protease is colored brightorange, 11a is blue (PDB: 6LZE). **(C)** The crystal structure of 11b-main protease complex. The main protease is brightorange, 11b is red (PDB: 6M0K). **(D)** The crystal structure of Carmofur-main protease complex. The main protease is brightorange, carmofur is cyan (PDB: 7BUY).

A mechanism-based inhibitor, N3, which was identified by the drug design aided by computer, could fit inside the substrate-binding pocket of the main protein and is a potent irreversible inhibitor of the main protein. Two of the Mpro-N3 complex associate to form a dimer (the two complexes are named protomer A and protomer B, respectively). Each protomer contains three domains which are designated as domain I−III. Both domain I and domain II have a β-barrel structure arranged in antiparallel manner. Domain III has five α-helices which associate to form a globular cluster structure in antiparallel manner. Domain III connects to domain II with a long loop. The cleft between domain I and domain II contains the substrate binding site. The backbone atoms of the compound N3 form an antiparallel sheet with residues 189–191 of the loop that connects domain II and domain III on one side, and with residues 164–168 of the long strand (residues 155–168) on the other (Jin et al., 2020a) (**Figure 5A**).

### 11a and 11b

Two compounds, namely 11a and 11b which target the M^pro^, exhibit excellent inhibitory effects on SARS-CoV-2 infection *in vitro*. The inhibitory activity of 11a and 11b at 1 µM is 100 and 96%. In vivo, the 11a and 11b exhibit good pharmacokinetics (PK) properties. Of note, 11a showed low toxicity as well. The -CHO group of 11a and 11b bond to the cysteine 145 of M^pro^ covalently. Different parts of 11a (designated as P1’, P1, P2, and P3) fits into different parts of the substrate-binding site. The (S)-γ-lactam ring of 11a at P1 inserts into the S1 site. The cyclohexyl moiety of 11a at P2 fits into the S2 site. At the part P3 of 11a, the indole group is exposed to the S4 site (in the solvent). The oxygen atom of -CHO forms a hydrogen bond with the cysteine 145 in the S1’ site. In addition, many water molecules (designated as W1−W6) are critical for binding 11a. The SARS-CoV-2 M^pro^-11b complex is similar to the SARS-CoV-2 M^pro^-11a complex and the 11a and 11b exhibit similar inhibitor binding mode (Dai W. et al., 2020) (**Figures 5B, C**).

### Camostat Mesylate

TMPRSS2 and TMPRSS4 are two mucosa-specific serine proteases which facilitate the fusogenic activity of SARS-CoV-2 spike protein and facilitate the virus to enter host cells (Zang et al., 2020). SARS-CoV-2 employs the TMPRSS2 in cells to prime the spike protein. TMPRSS2 activity is critical for the spread of SARS-CoV-2 as well as the pathogenesis in the infected host. Therefore, TMPRSS2 is a potential antiviral target. The spectrum of cell lines mediated entry by the S protein of SARS-CoV-2 and SARS-CoV are similar. Camostat mesylate, a clinical TMPRSS2 inhibitor, can partially block SARS-CoV-2 spike-driven entry into lung cells. In addition, camostat mesylate exhibits potent inhibit activity on SARS-CoV, SARS-CoV-2, and MERS-CoV, inhibiting them from entering lung cell line Calu-3, without cytotoxicity. In conclusion, camostat mesylate has the potential to treat and prevent COVID-19 (Hoffmann et al., 2020).

### Carmofur

The antineoplastic drug carmofur can inhibit the main protease (M^pro^) of SARS-CoV-2. The crystal structure of carmofur-main protease complex has been solved. Carmofur inhibits the activity of SARS-CoV-2 main protein *in vitro* and the half-maximum inhibitory concentration (IC50) is 1.82 μM. Carmofur is an approved antineoplastic agent used for colorectal cancer. It is a derivative of 5-fluoroyracil (5-FU). The molecular details of how carmofur inhibits the activity of SARS-CoV-2 main protein have not been resolved. One study showed the crystal structure of SARS-CoV-2 M^pro^-carmofur complex. The electron density figure indicates that the fatty acid moiety (C_7_H_14_NO) of carmofur links with the Sγ atom of SARS-CoV-2 main protein catalytic residue Cys145 covalently. The electrophilic carbonyl group of carmofur is attacked by the sulfhydryl group of Cys145. This process modifies the Cys145 covalently and releases the 5-FU motif. Notably, numerous hydrogen bonds and hydrophobic interactions stabilize the inhibitor carmofur. The fatty acid tail of carmofur (an extended conformation) inserts into the S2 subunit of SARS-CoV-2. Most of the hydrophobic interactions are contributed by His41, Met165, and Met49 in the side chain (Jin et al., 2020b) (**Figure 5D**).

### Lipopeptide EK1C4

The complex (6-HB) formed by the HR1 and HR2 of the SARS-CoV-2 S protein could facilitate the infection of the viruses (Xia et al., 2020b). EK1 is one kind of coronavirus fusion inhibitor and has an inhibitory effect on various coronaviruses. It targets the HR1 of the S protein of human coronavirus and has been proved to effectively inhibit the infection of five HCoVs, including SARS-CoV and MERS-CoV. Peptide EK1 could intervene the formation of viral 6-HB (Xia et al., 2020a). A recent study shows that the peptide EK1 could also inhibit the membrane fusion mediated by SARS-CoV-2 spike protein as well as SARS-CoV-2 pseudovirus infection in a dose-dependent manner (Xia et al., 2020a; Xia et al., 2020b). EK1C is constructed by covalently attaching the cholesterol acid to the C-terminal of EK1 sequence. It is noteworthy that the lipopeptide EK1C4 has the strongest inhibitory effect on the membrane fusion which is mediated by the spike protein, with IC50 of 4.3 nM. However, the IC50 of EK1 is 409.3 nM. EK1C4 could also potently inhibit the infection caused by live coronavirus *in vitro* with little, or even no, toxic effect. In conclusion, EK1C4 has the potential to be used for the treatment and prevention of COVID-19 (Xia et al., 2020a).

## Vaccines of SARS-CoV-2

It is urgent to develop effective and safe vaccines to control the new occurrence of COVID-19 and to reduce SARS-CoV-2-infection-related morbidity and mortality (Amanat and Krammer, 2020). Chinese Health Commission announced that more than five kinds of vaccines are currently developed for COVID-19 in China, including subunit protein vaccine, nucleic acid vaccine, inactivated vaccine, adenoviral vector vaccine, and influenza viral vector vaccine (Lu, 2020; Sun J. et al., 2020). As of October 17, 2020, there are 177 vaccine candidates for COVID-19 and 54 are in human trials in the world (https://biorender.com/covid-vaccine-tracker). For example, the non-replicating Ad5 vectored COVID-19 vaccine produced by CanSino Biologics lnc, the mRNA-1273 COVID-19 vaccine developed by Moderna, the DNA vaccine of Inovio Pharmaceuticals, the BioNTech’s mRNA COVID-19 vaccine, the vaccine ChAdOx1 nCoV-19 of University of Oxford (Zhu et al., 2020), the adenovirus serotype 26 vector-based vaccine Ad26.COV2.S, the Novavax’s protein subunit vaccine NVX-CoV2373, the Sinovac’s inactive vaccine CoronaVac, the Chulalongkorn University’s mRNA vaccine ChulaCov19, etc. (https://biorender.com/covid-vaccine-tracker). Currently, clinically approved vaccines are not widely available (Hu B. et al., 2020). The safety and efficacy of the vaccines should be kept in mind in the efforts of vaccine development. Following are some notable SARS-CoV-2 vaccines in development.

### mRNA-1273

Moderna’s mRNA-based vaccine stimulates the expression of target antigen after injection of mRNA encapsulated in nanoparticles (Amanat and Krammer, 2020). The vaccine is called mRNA-1273, it is a synthetic mRNA strand, which can encode the viral spike protein that is stable before fusion. After being injected into the body intramuscularly, the vaccine mRNA-1273 could stimulate antiviral response that targets the spike protein of SARS-CoV-2 specifically. Different from conventional route of vaccine development, the lipid mRNA nanoparticle-encapsulated mRNA vaccine can be synthesized and made without the virus (Tu et al., 2020). At present, mRNA-1273 has completed phase I clinical trial (ClinicalTrials.gov Identifier: NCT04283461) and phase II clinical trial. The results of the mRNA-1273 vaccine phase I clinical trial in 45 healthy adults (18–55 years old) show a strong antibody and cellular immune response in participants and no safety concerns are identified (Jackson et al., 2020). Phase II clinical trial is a dose-conformation study used to evaluate the safety, reactogenicity, and immunogenicity of mRNA-1273 in healthy adults. The phase III clinical trial has started on July 27, 2020 (ClinicalTrials.gov Identifier: NCT04470427). This is a randomized, stratified study to evaluate the efficacy, immunogenicity, and safety of the vaccine in healthy adults.

### Recombinant Adenovirus Type-5 (Ad5) Vectored Vaccine

The phase I clinical trial of an Ad5 vectored COVID-19 vaccine has been done in Wuhan, China. The Ad5 vectored COVID-19 vaccine targets the spike protein of SARS-CoV-2. This trial is a dose-escalation, non-randomized, open-label, and first-in-human trial. The vaccine trial had three dose groups, including 5×10¹⁰, 1×10¹¹, and 1.5×10¹¹ viral particles. A total of 108 participants who were healthy and aged between 18−60 years old were allocated to one of the three dose group and each group contains 36 participants. The vaccine is injected intramuscularly into the human body. Results indicated that participants in all the dose groups exhibited at least one adverse reaction within 7 days post-vaccination. The most reported adverse reaction at the injection site was pain. Fever and fatigue were the most common systematic symptoms, 46 and 44% of the recipients exhibited such symptoms, respectively. However, most reported adverse reactions were mild or moderate in severity. Within 28 days after vaccination, no serious adverse reactions were reported. Humoral responses against SARS-CoV-2 peaked 28 days after vaccination in participants. From 14 days after vaccination, the specific T-cell responses were notable and rapid. Results demonstrate that this vaccine is immunogenic and tolerable in healthy adults and has the potential to control the outbreak of COVID-19. However, further investigations are needed to identify the immunogenicity and safety of this vaccine (Zhu et al., 2020). The phase II trial in China (NCT04341389) has started. This is a randomized, double-blinded and placebo-controlled clinical trial in healthy adults. The purpose of the study is to evaluate the safety and immunogenicity of Ad5 vectored vaccine.

### PiCoVacc

In a recent study, a purified inactivated SARS-CoV-2 virus vaccine candidate (PiCoVacc) is developed in a pilot-scale production. The target of PiCoVacc is the entire virus. The study indicated that PiCoVacc could induce neutralizing antibodies which neutralized 10 representative SARS-CoV-2 strains in mice, rats, and non-human primates, suggesting its strong potential to neutralizing the other SARS-CoV-2 strains that are circulating. Six μg per dose of the PiCoVacc could protect the macaques from SARS-CoV-2 infection completely and systematic evaluation suggests its safety (Gao Q. et al., 2020).

### DNA Vaccines

A recent study (Yu J. et al., 2020) has produced a series of DNA vaccine candidates which express six variants of the spike protein of the SARS-CoV-2. DNA vaccines targets the spike protein of SARS-CoV-2. The candidates were evaluated in 35 rhesus macaques. At week 0 and week 3, rhesus macaques were injected 5 mg DNA vaccines intramuscularly. S-specific binding antibodies and neutralizing antibodies (NAbs) were detected after the boost immunization at week 5. Neutralizing antibody (NAb) titers in the vaccinated macaques were comparable to the Nab titers in 9 convalescent rhesus macaques and 27 convalescent patients who were infected with SARS-CoV-2. Cellular immune responses targeting the S peptides were observed in most of the vaccinated rhesus macaques at week 5. At week 6, all rhesus macaques were challenged with 1.2×10^8^ VP SARS-CoV-2 intranasally and intratracheally. Compared to the control groups, lower levels of SARS-CoV-2 RNA were observed in the vaccine groups. Reduced levels of subgenomic mRNA (sgmRNA) in bronchoalveolar lavage (BAL) and nasal swabs (NS) were observed in vaccine groups. In conclusion, these DNA vaccines prevent rhesus macaques from being infected by SARS-CoV-2 and may accelerate the development of SARS-CoV-2 vaccine which are urgently needed to protect humans from SARS-CoV-2 infections.

### A Universal Betacoronavirus Vaccine Against COVID-19, MERS, and SARS

The RBD of coronaviruses is an attractive vaccine target. However, RBD-based vaccines have relatively low immunogenicity. One study describes the dimeric form of MERS-CoV RBD. Compared to monomeric form, the RBD-dimer could expose double receptor-binding motifs and increase neutralizing antibody (NAb) titers significantly, so as to overcome the limitation of low immunogenicity. RBD-sc-dimer is a stable version of RBD-dimer with high vaccine efficacy. When using this strategy to design vaccines against SARS and COVID-19, 10–100-fold enhancement of Nab titers were achieved. Notably, the Nab titers caused by two-dose of RBD-sc-dimer is much higher than the RBD-sc-dimer, reaching ~4,096 (Dai L. et al., 2020).

On June 23, 2020, the clinical phase III trial of the inactivated SARS-CoV-2 vaccine developed by the SINOPHARM CNBG launched officially. This is the first international clinical phase III trial of inactivated SARS-CoV-2 vaccine. The clinical phase III trial takes about half a year to evaluate the safety and effectiveness of the vaccine in a larger population.

## Discussion

The rapid global pandemic of SARS-CoV-2 has already posed a great threat to human health, social health system, global economy, and even the global governance, and these influences may likely continue for a longer time. We need to learn the epidemic logic of SARS-CoV-2 and study the unknown viruses carried by wild animals in nature in advance to make early warning. There may be an outbreak caused by other kinds of viruses next time except for SARS-CoV-2. Study experiences and lessons on different viruses may be referenced each other.

Sufficient understanding on the differences of structural and non-structural proteins among SARS-CoVs and other coronaviruses may help to the development of therapeutics for SARS-CoV-2 infection. The non-structural proteins of the coronaviruses which can infect humans are relatively similar with SARS-CoV-2 in structure. Except S protein, most of the structural proteins, such as E protein and M protein, showed no significant difference in protein architecture between SARS-CoV-2 and other known human CoVs. The S protein of CoVs is responsible for binding the host cell-surface receptor during host cell entry. Different CoVs recognize different cell surface receptor. For instance, MERS-CoVs recognize the dipeptidyl peptidase 4 receptor. Nevertheless, SARS-CoV and SARS-CoV-2 recognize the ACE2 receptor. This may be the reason why different CoVs have various host entry mechanism. In addition, the SARS-CoV-2 RBD has higher hACE2 binding affinity than SARS-CoV RBD, supporting efficient cell entry. Unlike SARS-CoV, the entry of SARS-CoV-2 is preactivated by proprotein convertase furin, reducing its dependence on target cell proteases for entry. The high hACE2 binding affinity of the RBD and the furin preactivation of the spike allow SARS-CoV-2 to maintain efficient cell entry while evading immune surveillance. These features may contribute to the wide spread of the virus.

Due to the limited knowledge about SARS-CoV-2 at present, there is no approved therapeutic drugs or vaccines available for the treatment of COVID-19. As the global epidemic worsens, effective and safe vaccines, antibodies, and specific anti-SARS-CoV-2 drugs are in urgent need. Neutralizing antibodies are expected effective for SARS-CoV-2 infection, but their costs, production scales, and covering rates are still questions. Vaccine development also faces difficulties such as timeliness and ineffectiveness due to inconsiderate vaccine design and/or virus mutation.

Unlike the vaccine which has a relatively clear mechanism and route in development, antiviral drugs with high potency and high safety may be more difficult to develop at present because of our incomplete understanding on the virus world and the host responsiveness. This may be the reason why there is no one satisfactory antiviral drug available till now. The potential *in vivo* toxic effect of an antiviral drug which is claimed effective *in vitro* may disappointingly overwhelm its pharmacological applause. New and unconventional ideas and routes of antiviral drug design and development are needed to overcome the shortcomings of the present reductionism-based drug research. In this aspect, the holism-based traditional Chinese medicine especially the Chinese medical herb formulae could be considered which are proved effective in the fight against COVID-19 in China (Ang et al., 2020; Luo et al., 2020; Ren et al., 2020; Zhang et al., 2020a; Zhong et al., 2020), although further mechanistic studies and large-scale clinical trials are warranted.

Recently, many countries in the world have increased their investment in the research and development of antiviral drugs, antibodies, and vaccines. Accelerating the development of vaccines is likely the current keyway to solve this global disaster. Through joint efforts around the world, people can develop effective anti-SARS-CoV-2 technologies hopefully in the near future.

## Author Contributions

M-YW and RZ wrote the manuscript. L-JG and X-FG helped drew the figures. D-PW and J-MC revised the manuscript. All authors contributed to the article and approved the submitted version.

## Funding

This work and related studies were supported by Shanxi “1331 Project” Key Subjects Construction (1331KSC), Applied Basic Research Program of Shanxi Province (201801D221269), Scientific and Technological Innovation Programs of Higher Education Institutions in Shanxi (STIP) (2019L0437), and the National Natural Science Foundation of China (81670313).

## Conflict of Interest

The authors declare that the research was conducted in the absence of any commercial or financial relationships that could be construed as a potential conflict of interest.
